# Patterns and Determinants of Double-Burden of Malnutrition among Rural Children: Evidence from China

**DOI:** 10.1371/journal.pone.0158119

**Published:** 2016-07-08

**Authors:** Nan Zhang, Laia Bécares, Tarani Chandola

**Affiliations:** 1 Cathie Marsh Institute for Social Research (CMIST), School of Social Sciences, The University of Manchester, Manchester, M13 9PL, United Kingdom; 2 Centre on Dynamics of Ethnicity (CoDE), School of Social Science, The University of Manchester, Manchester, M13 9PL, United Kingdom; Institute for Health & the Environment, UNITED STATES

## Abstract

Chinese children are facing dual burden of malnutrition—coexistence of under-and over-nutrition. Little systematic evidence exists for explaining the simultaneous presence of under-and over-nutrition. This study aims to explore underlying mechanisms of under-and over-nutrition among children in rural China. This study used a nationwide longitudinal dataset of children (*N* = 5,017) from 9 provinces across China, with four exclusively categories of nutritional outcomes including under-nutrition (stunting and underweight), over-nutrition (overweight only including obesity), paradox (stunted overweight), with normal nutrition as reference. Multinomial logit models (Level-1: occasions; Level-2: children; Level-3: villages) were fitted which corrected for non-independence of observations due to geographic clustering and repeated observations of individuals. A mixture of risk factors at the individual, household and neighbourhood levels predicted under-and over-nutrition among children in rural China. Improved socioeconomic status and living in more urbanised villages reduced the risk of stunted overweight among rural children in China. Young girls appeared to have higher risk of under-nutrition, and the risk decreased with age more markedly than for boys up to age 5. From age 5 onwards, boys tended to have higher risk of under-nutrition than girls. Girls aged around 12 and older were less likely to suffer from under-nutrition, while boys’ higher risk of under-nutrition persisted throughout adolescence. Children were less likely to suffer from over-nutrition compared to normal nutrition. Boys tended to have an even lower risk of over-nutrition than girls and the gender difference widened with age until adolescence. Our results have important policy implications that improving household economic status, in particular, maternal education and health insurance for children, and living environment are important to enhance rural children’s nutritional status in China. Investments in early years of childhood can be effective to reduce gender inequality in nutritional health in rural China.

## Introduction

Economic growth is associated with improved child nutrition [1], and China is no exception. The nutritional status of Chinese children has steadily improved in the last few decades [2–7]. Evidence from nationally representative datasets [8,9] and from the nationwide data China Health and Nutrition Survey (CHNS) [4] consistently show that the prevalence of under-nutrition such as stunting and underweight among children in China has been substantially reduced. Although a remarkable improvement of childhood nutrition in China has been achieved, there remains considerable under-nutrition, including stunted growth and underweight, among children in poor regions and rural areas [8,10–13]. Moreover, new and worrisome issues have emerged, such as the coexistence of under-and over-nutrition [14–16], and the increasing prevalence of overweight and obesity, particularly in urban areas and affluent rural areas [11,17–19].

The association between stunting and overweight among children has been observed in societies undergoing a nutrition transition, referring to changes in diet composition from traditional diets low in fat and high in fibre to more ‘Western’ diets high in energy and low in fibre, which is closely linked to a process of rapid socio-economic development and urbanisation, such as China [20,21], and some other low- to middle-income countries [22]. Popkin [20] has argued that the coexistence of stunting and overweight among children did not emerge apparently prior to the shift in incomes and the related lifestyle in most low income countries, where stunted children had little opportunity, in terms of economic conditions and food resources available, to become obese. Evidence has suggested that among nutritionally stunted children, adaption to nutritional insults that affect enzyme and hormone function is associated with impaired fat oxidation [23], this has been shown to be a risk factor for excessive weight gain [24,25]. Children who are stunted and overweight simultaneously can be at greater risk of unhealthy development than other children. Furthermore, both childhood obesity and short stature are risk factors for the metabolic syndrome and Type 2 diabetes in adulthood [26,27]. Therefore, the combination of stunting and obesity during childhood can lead to both undernutrition-related diseases, infectious diseases and obesity-related diseases, resulting in double burden of diseases in China.

Despite a number of studies, in particular cross-sectional studies that suggest an increased prevalence of over-nutrition and decreased prevalence of under-nutrition among children, results are limited by their restricted generalisability, as the sample is restricted to certain age ranges (mostly conducted among children under 5 years) [28–30], to their geographical location [30–35], and to the relatively short study periods assessed to date, which may fail to capture temporal and spatial dynamics of children’s nutritional transition in China with rapid social and economic change.

Previous studies have used inclusive measures of child malnutrition (such as overweight, stunting, and underweight), which may fail to capture the severity of malnutrition for some children who suffer from more than one malnutrition status. For example, Wang et al. (2009) argued that the relatively high prevalence of overweight among rural children could be a false impression, suggesting that many ‘overweight’ children are actually stunted. This could be described in a separate category as ‘stunting overweight’ [28], not really ‘overweight’. In addition, given the specific cultural context of rural China where son preferences are prevalent [36], the present study includes a special focus on examining how gender is related to the prevalence of under- and-over nutrition, as well as how these relationships change as children age. The gender of children is relevant because boys and girls differ in energy and nutrient needs [37], but also because of gender discrimination in favour of boys in intra-household allocation in rural China [36]. It is important to adopt a life course approach to examine how the relationship between gender and malnutrition vary with age for two reasons. One the one hand, children of both sexes experience dissimilar growth rates when they get older [37]. On the other hand, a life course approach recognises the importance of time (age) and timing (during particular age periods) in understanding the associations between exposure (e.g., gender) and outcomes (e.g., child malnutrition) within an individual life course [38]. This approach, for example, can depict when and how gender differences in child malnutrition diverge or converge during childhood, which can facilitate interventions, targeted at specific age ranges in order to reduce gender inequality in rural China.

Based on the arguments above, studies reporting the degree of overweight/obesity among rural children in China should be interpreted with caution. Thus, this study focuses on exclusive measures of child malnutrition in terms of under-nutrition (stunting and/or underweight), over-nutrition (overweight and obesity only) and paradox (stunted overweight), Drawing on nationwide longitudinal datasets, this study aims to identify factors that determine exclusive malnutrition status among children in rural China, and whether these associations differ between boys and girls when they get older.

## Subjects and Methods

### Dataset

Data were drawn from the China Health and Nutrition Survey (CHNS), an ongoing open-cohort study which employs a multistage, random-clustered sampling process to draw a sample of around 4,400 households with a total of about 19,000 participants from over 200 communities or neighbourhoods in nine provinces, with the first round conducted in 1989. The CHNS covers nine provinces that vary substantially in geography, economic development, public resources, and health indicators. The design, sampling, and response rates are reported elsewhere [39].

The present study used data from five waves of the CHNS, collected in 1991, 1993, 1997, 2000, 2004, 2006, and 2009. Children less than18 years old whose household registration type (*hukou*) was classified as ‘rural’ in the CHNS dataset were included. The institutional review committees from the University of North Carolina at Chapel Hill and the National Institute for Nutrition and Food Safety, China Centre for Disease Control and Prevention, approved the survey protocols and instruments and the process for obtaining informed consent for this survey. All participants and/or their parents/guardians provided written informed consent for their participation in the survey.

### Outcomes of interest

The CHNS recorded height and weight for each individual within the household, measured by health professionals. There are mainly three anthropometric measurements for children’s nutritional status, including height-for-age Z-scores (HAZ), weight-for-age Z-scores (WAZ), and Body-mass-index Z-scores (BMIZ). Z-scores are used to compare children’s nutritional status across age and gender [40]. HAZ scores are an indicator of chronic malnutrition indicating cumulative long-term health status, whereas weight-for-height Z-scores (WHZ) indicate more recent short-term deprivation. WAZ scores to some extent confound the two (short term and cumulative nutrition), and are mainly affected by acute socio-economic factors [41]. WHZ scores were not reported in this study because the short-term changes reflected by WAZ scores could be empirically indistinguishable from variations due to a longitudinal study design of the CHNS.

Z scores were constructed with the Chinese growth reference which are based on Chinese urban children [42]. Children whose HAZ scores or WAZ scores are below -2 (i.e., more than 2 standard deviations below the reference children’s median height and weight), are classified to be *stunting* or *underweight*, respectively. BMIZ scores below -2 and over 1 are defined as *thinness* (distinguished from *underweight* measured by WAZ) and *overweight* (including *obesity*). *Thinness* was collapsed into *underweight* group.

Given different dimensions and potential overlap of nutritional status (for example, stunting can coexist with underweight or with overweight), we created an exclusive measure of nutritional status—malnutrition, which has six categories: 1-normal (normal weight and not stunting) as reference group, 2-stunting only (normal weight and stunting), 3-overweight only (overweight but not stunting), 4-stunted overweight, 5-underweight only (underweight but not stunting), and 6-stunting underweight. Table 1 shows sample sizes and distributions of six scenarios of malnutrition among children by age groups in China.

**Table 1:** Sample sizes and percentages of stunting across overweight/underweight status among children in rural China, the CHNS 1991–2009.

	Overweight/underweight status			Total
Nutritional status	Normal	Overweight	Underweight	
Not stunted	6,603(56.78)	1,111(9.55)	174(1.27)	7,888(67.82)
Stunted	2,417(20.78)	589(5.06)	736(6.33)	3,742(32.18)
Total	9,020(77.56)	1,700(14.62)	990(7.82)	11,630(100)

CHNS, China Health and Nutrition Survey

Due to relatively small sample sizes in certain scenarios of malnutrition, for instance, ‘underweight only’ and ‘stunting underweight’, we further collapsed ‘stunting only’ ‘underweight only’ and ‘stunting underweight’ into one category named as ‘under-nutrition’. Accordingly, ‘overweight only’ can be considered as ‘over-nutrition’, and ‘stunted overweight’ as a paradox of under-and-over nutrition (Table 2). Doing so provided sufficient sample sizes to run multinomial logit models in the remainder of this study.

**Table 2:** Sample sizes and percentages of four exclusive malnutrition categories among children in rural China, the CHNS 1991–2009.

Malnutrition status	Normal	Under-nutrition (stunted and/or underweight)	Over-nutrition (overweight only)	Paradox (stunted overweight)	Total
**N (%)**	6,603(56.78)	3,327(28.61)	1,111(9.55)	589(5.06)	11,630(100)

CHNS, China Health and Nutrition Survey

### Variables

#### Individual/household-level risk factors

Individual-level variables included maternal education (the number of years of formal education completed), and annual household income per capita at the household level. Individual-level variables included only child (whether a child is the only child within a household), and child insurance (whether a child has health insurance or not). In addition, we controlled for socio-demographic variables including age and gender. To control for differential purchasing power across the households, household income per capita was categorised into tertiles of low, medium, and high income groups.

#### Neighbourhood-level risk factors

An urbanisation index was produced for each community (village) according to a range of village-level data from the CHNS including population density, transportation infrastructure, sanitation, housing, health infrastructure and some other factors that can distinguish features of urban places [43]. This variable was adjusted to take into account environmental risk factors for children’s nutritional status [44,45]. To control for neighbourhood effects on childhood nutritional status, we focused on relative urbanisation rankings by creating urbanisation tertiles for each village.

Additionally, we created regional dummy variables according to the geographical and socio-economic disparities: coastal (Shandong and Jiangsu, the two most economically developed provinces), northeast (Liaoning and Heilongjiang), central inland (Henan, Hubei and Hunan), and the mountainous south (Guangxi and Guizhou), the most economically deprived region, was set as the reference region. Regional dummies were controlled in order to capture unobserved geographic and cultural factors related to food consumption and food prices, which may influence children’s nutritional status. We also included wave dummies to capture temporal effects with 1991 as reference.

### Statistical analysis

Unordered logistic regressions modelling techniques were used due to the nominal nature of the dependent variables. To account for the hierarchical structure of the data, a multilevel approach was adopted. Due to the relatively small number of children within the same household on average, we ignored household level and focused on the village level and child level. Therefore, the models were fitted with repeated measurements at Level-1, individuals at Level-2 and villages at Level-3. This corrects the estimated standard errors, thereby handling the clustering of observations that occurs within units [46].

Multinomial logit models were conducted within MLwiN 2.28 [47] for four-category outcomes of 0: normal nutritional status (reference category) without any malnutrition status, 1: suffering from under-nutrition (stunting and underweight), 2: suffering from over-nutrition (overweight only including obesity), and 3: suffering from ‘paradox’ of malnutrition (stunted overweight). Formally, *Y*_*ijk*_ is the categorical outcome with *t* categories for the *j*th (*j* = 1,2,…,N) child at occasion *i* (*i* = 1, 2,…,T) in village *k* (*k* = 1, 2,…, K). We denoted the probability of being in category *s* taking the following form:

$$\pi_{ijk}^{\left(s\right)}=Pr\left(Y_{ijk}=s\right)$$ (1)

We estimated a set of (*t*-1) logistic regressions for under-nutrition, over-nutrition, and ‘paradox’ in which each of the categories was contrasted with the reference category.

Then, a multilevel multinomial logistic regression model with logit link can be written as:

$$log\left(\frac{\pi_{ijk}^{\left(s\right)}}{\pi_{ijk}^{\left(t\right)}}\right)=\;\beta_{0}^{\left(s\right)}+\beta_{1}^{\left(s\right)}X+\left(v_{jk}^{\left(s\right)}+f_{k}^{\left(s\right)}\right)$$ (2)

where *s* = 1,…, *t*-1. A separate intercept and slope parameter was estimated for over-nutrition, under-nutrition, and the ‘paradox’ of malnutrition (stunted overweight) as denoted by the *s* superscripts. $\beta_{0}^{\left(s\right)}$ is the logarithm of the ratio of the overall probability of a child being over-nutrition, under-nutrition, or ‘paradox’ of malnutrition in category *s* to the reference category.

*X* is a set of predictor variables including socio-demographic and socio-economic predictors at different levels. $\beta_{1}^{\left(s\right)}$ represents the fixed parts of the model and is interpreted as the effect of a 1-unit increase in *X* on the logs odds of being in category *s* (i.e., over-nutrition, under-nutrition or ‘paradox’) rather than category *t* (usually the reference category). For presentations and discussion, we used exp($\beta_{0}^{\left(s\right)}$), which is the effect of a 1-unit increase *X* on the odds of being in category *s* rather than category *t*. $\left(v_{jk}^{\left(s\right)}+f_{k}^{\left(s\right)}\right)$ refer to random effects representing level-2 (children) and level-3 (villages) residuals, respectively. They are assumed to be normally distributed with mean 0 and variances $\delta_{v}^{2\left(s\right)}$ and $\delta_{f}^{2\left(s\right)}$. The random effects are specific to each of the contrasted categories as denoted by the *s* superscripts, because different unobserved child-level (level-2) and village-level (level-3) factors may affect each contrast outcome. Moreover, the random effects may be correlated across contrasts: $\text{Cov}(v_{jk}^{\left(s\right)}+v_{jk}^{\left(r\right)})\;=\;\delta_{v}^{\left(s,\;r\right)}$ for child-level covariance, and $\text{Cov}(f_{k}^{\left(s\right)}+f_{k}^{\left(r\right)})\;=\;\delta_{f}^{\left(s,\;r\right)}$ for village-level covariance, s ≠ r. Correlated random effects would arise, for example, if there are unobserved village-level factors (e.g., food environment and eating patterns) which affect the choice of more than one category of malnutrition. In order to examine gender differences in malnutrition status when children grow up, we also fitted models that included the interaction of age and gender for children in the model.

The multilevel models in this study were estimated using Bayesian methods implemented via Markov Chain Monte Carlo (MCMC) simulation [48]. MCMC estimates were employed to reduce bias in the estimates of the random effects parameters, which can arise when multilevel models with discrete outcomes are estimates using maximum-likelihood procedures [49].

## Results

The sample was restricted to complete cases for the nutritional outcomes and predictors considered in the analyses. The sample size of children under 18 years of age was 5,017 clustered in 177 villages with 11,630 observations.

Table 3 summarises samples and distributions of children across four categories of malnutrition by different predictors from the CHNS 1991–2009. Boys were more likely to suffer from under-nutrition and paradox (hereby stunted overweight) than girls, while boys were less likely to suffer from over-nutrition than girls. Household economic status is an important predictor that affect the distributions of childhood malnutrition; children from the highest income families were more likely to suffer from over-nutrition and less likely to suffer from under-nutrition and stunted overweight. Children from the least urbanised villages had the highest prevalence of being stunted overweight as well as under-nutrition. The most urbanised villages tended to have lowest risk of malnutrition in terms of under-nutrition, over-nutrition and stunted overweight. There were substantial regional and temporal variations in childhood malnutrition. The mountainous southern areas, the most deprived of the survey regions, had the highest prevalence of under-nutrition and the lowest over-nutrition prevalence. The prevalence of under-nutrition and stunted overweight among rural children decreased over time from 1991 to 2009.

**Table 3:** Sample size and the distribution of children across four categories of malnutrition by different predictors from the CHNS 1991–2009.

		Malnutrition with four scenarios			
Variables	Sample sizes	Normal	Under-nutrition(stunting & underweight)	Over-nutrition(Overweight only)	‘Paradox’(Stunted overweight)
Sample size	11,630	6,603	3,327	1,111	589
	*n(%)*	*n(%)*			
Gender***					
Boy	6,223 (53.51)	3,517 (53.26)	1,874 (56.33)	523 (47.07)	309 (52.46)
Girl	5,407 (46.49)	3,086 (46.74)	1,453 (43.67)	588 (52.93)	280 (47.54)
Only child***					
No	9,456 (81.31)	5,256 (79.60)	2,901 (87.20)	811 (73.00)	488 (82.85)
Yes	2,174 (18.69)	1,347 (20.40)	426 (12.80)	300 (27.00)	101 (17.15)
Health insurance***					
No	8,752 (75.25)	4,767 (72.19)	2,772 (83.32)	740 (66.61)	473 (80.31)
Yes	2,878 (24.75)	1,836 (27.81)	555 (16.68)	371 (33,39)	116 (19.69)
Household income per capita***					
Lowest	4,667 (40.13)	2,379 (36.03)	1,610 (48.39)	395 (35.55)	283 (48.05)
Middle	3,816 (32.81)	2,161 (32.73)	1,142 (34.33)	315 (28.35)	198 (33.62)
Highest	3,147 (27.06)	2,063 (31.24)	575 (17.28)	401 (36.09)	108 (18.34)
Urbanisation score***					
Lowest	5,295 (45.53)	2,731 (41.36)	1,773 (53.29)	445 (40.05)	346 (58.74)
Middle	4,699 (40.40)	2,819 (42.69)	1,258 (37.81)	440 (39.60)	182 (30.90)
Highest	1,636 (14.07)	1,053 (15.95)	296 (8.90)	226 (20.34)	61 (10.36)
Region***					
South	3,947 (33.94)	1,870 (28.32)	1,694 (50.92)	189 (17.01)	194 (32.94)
Coastal	1,836 (15.79)	1,081 (16.37)	313 (9.41)	299 (26.91)	143 (24.28)
Northeast	1,607 (13.82)	1,156 (17.51)	183 (5.50)	221 (19.89)	47 (7.98)
Central	4,240 (36.46)	2,496 (37.80)	1,137 (34,17)	402 (36.18)	205 (34.80)
Wave***					
1991	2,236 (19.23)	1,040 (15.75)	885 (26.60)	159 (14.31)	152 (25.81)
1993	2,406 (20.69)	1,233 (18.67)	818 (24.59)	195 (17.55)	160 (27.16)
1997	1,889 (16,24)	1,094 (16.57)	575 (17.28)	141 (12.69)	79 (13.41)
2000	1,747 (15.02)	1,087 (16.46)	450 (13.53)	143 (12.87)	67 (11.38)
2004	1,263 (10.86)	790 (11.96)	260 (7.81)	166 (14.94)	47 (7.98)
2006	1,039 (8.93)	659 (9.98)	189 (5.68)	145 (13.05)	46 (7.81)
2009	1,050 (9.03)	700 (10.60)	150 (4.51)	162 (14.58)	38 (6.45)

*Chi-square test* for a cross-tabulation between each variable and the four categories of malnutrition:

*p<0.05,

**p<0.01,

***p<0.001.

CHNS, China Health and Nutrition Survey

Tables 4 and 5 present the estimated adjusted relative odds ratios (RRR) with their 95% confidence intervals (CI) from multinomial logit models on malnutrition with four scenarios (using normal nutrition without being stunting, underweight or overweight as the reference) with and without the interaction of age and gender for children less than 18 years in rural China.

**Table 4:** Adjusted relative risk ratios (RRR) and 95% confidence intervals (CI) for predictors of unordered multinomial logistic models [using normal nutrition without being stunted, underweight or overweight as the reference] predicting malnutrition for children less than 18 years from the CHNS, 1991–2009.

Fixed effects	Malnutrition (reference: normal nutrition)		
	Under-nutrition(stunting and underweight)	Over-nutrition(overweight only)	‘Paradox’(stunted overweight)
	RRR (95% CI)	RRR (95% CI)	RRR (95% CI)
Age (years)	0.93 (0.91, 0.94)	0.84 (0.82, 0.86)	0.74 (0.72, 0.76)
*p-value*	<0.001	<0.001	<0.001
Gender			
Boy (reference)			
Girl	0.69 (0.57, 0.84)	1.62 (1.35, 1.94)	0.99 (0.78, 1.26)
*p-value*	<0.001	<0.001	0.96
Only child within household			
No, having siblings (reference)			
Yes, only child	1.00 (0.82, 1.22)	1.23 (0.99, 1.52)	1.22 (0.90, 1.64)
*p-value*	0.99	0.06	0.20
Health insurance			
No (reference)			
Yes	0.77 (0.63, 0.94)	0.98 (0.80, 1.20)	0.76 (0.56, 1.03)
*p-value*	0.012	0.86	0.08
Household income per capita			
Lowest (reference)			
Middle	0.92 (0.79, 1.07)	0.88 (0.72, 1.08)	1.06 (0.83, 1.35)
Highest	0.73 (0.59, 0.90)	1.01 (0.80, 1.27)	0.77 (0.56, 1.05)
*p-value*	0.013	0.36	0.14
Maternal education (years)	0.94 (0.92, 0.97)	1.00 (0.97, 1.03)	0.96 (0.93, 1.00)
*p-value*	<0.001	0.98	0.06
Urbanisation index			
Lowest (reference)			
Middle	0.90 (0.76, 1.07)	0.96 (0.77, 1.20)	0.66 (0.50, 0.87)
Highest	0.67 (0.49, 0.90)	1.23 (0.89, 1.69)	0.65 (0.40, 1.05)
*p-value*	0.03	0.18	0.011
Region			
South (reference)			
Coastal	0.09 (0.06, 0,15)	5.08 (3.65, 7.08)	0.69 (0.36, 1.32)
Northeast	0.06 (0.04, 0.10)	2.70 (1.87, 3.88)	0.22 (0.12, 0.41)
Central	0.24 (0.17, 0.34)	2.12 (1.56, 2.88)	0.52 (0.31, 0.86)
*p-value*	<0.001	<0.001	<0.001
Wave			
1991 (reference)			
1993	0.69 (0.57, 0.83)	1.27 (0.98, 1.65)	1.08 (0.82, 1.44)
1997	0.50 (0.40, 0.63)	1.15 (0.86, 1.53)	0.69 (0.48, 1.00)
2000	0.41 (0.31, 0.53)	1.41 (1.04, 1.92)	0.68 (0.46, 1.01)
2004	0.26 (0.19, 0.36)	2.17 (1.60, 2.96)	0.45 (0.28, 0.71)
2006	0.20 (0.14, 0.28)	2.02 (1.45, 2.81)	0.39 (0.24, 0.63)
2009	0.13 (0.08, 0.20)	1.99 (1.35, 2.93)	0.27 (0.15, 0.48)
*p-value*	<0.001	<0.001	<0.001

Continuous predictors are centred on grand mean

CHNS, China Health and Nutrition Survey

**Table 5:** Adjusted relative risk ratios (RRR) and 95% confidence intervals (CI) for predictors of unordered multinomial logistic models [using normal nutrition without being stunted, underweight or overweight as the reference] with the interaction of age and gender predicting malnutrition for children less than 18 years from the CHNS, 1991–2009.

Fixed effects	Malnutrition (reference: normal nutrition)		
	Under-nutrition(stunting and underweight)	Over-nutrition(overweight only)	‘Paradox’(stunted overweight)
	RRR (95% CI)	RRR (95% CI)	RRR (95% CI)
Age (years)	0.96 (0.94, 0.98)	0.81 (0.79, 0.83)	0.74 (0.71, 0.77)
*p-value*	<0.001	<0.001	<0.001
Gender			
Boy (reference)			
Girl	1.54 (1.12, 2.11)	0.98 (0.73, 1.33)	1.11 (0.70, 1.75)
*p-value*	<0.01	0.91	0.66
Age*gender	0.92 (0.90, 0.95)	1.06 (1.03, 1.10)	1.01 (0.95, 1.06)
*p-value*	<0.001	<0.001	0.84
Only child within household			
No, having siblings (reference)			
Yes, only child	0.99 (0.80, 1.21)	1.26 (1.03, 1.55)	1.22 (0.90, 1.64)
*p-value*	0.91	0.03	0.20
Health insurance			
No (reference)			
Yes	0.78 (0.64, 0.95)	0.97 (0.78, 1.20)	0.77 (0.57, 1.02)
*p-value*	0.013	0.79	0.07
Household income per capita			
Lowest (reference)			
Middle	0.93 (0.80, 1.09)	0.89 (0.73, 1.08)	1.08 (0.85, 1.36)
Highest	0.74 (0.61, 0.89)	1.01 (0.79, 1.28)	0.79 (0.58, 1.07)
*p-value*	<0.01	0.39	0.12
Maternal education (years)	0.94 (0.92, 0.97)	1.00 (0.97, 1.03)	0.97 (0.93, 1.00)
*p-value*	<0.001	0.90	0.07
Urbanisation index			
Lowest (reference)			
Middle	0.90 (0.74, 1.09)	0.98 (0.79, 1.20)	0.67 (0.51, 0.89)
Highest	0.67 (0.49, 0.91)	1.22 (0.89, 1.68)	0.67 (0.43, 1.06)
*p-value*	0.04	0.25	0.02
Region			
South (reference)			
Coastal	0.10 (0.06, 0.17)	4.85 (3.23, 7.28)	0.72 (0.35, 1.45)
Northeast	0.07 (0.05, 0.11)	2.60 (1.73, 3.90)	0.25 (0.13, 0.47)
Central	0.28 (0.19, 0.41)	2.03 (1.41, 2.92)	0.59 (0.32, 1.08)
*p-value*	<0.001	<0.001	<0.001
Wave			
1991 (reference)			
1993	0.70 (0.58, 0.84)	1.23 (0.94, 1.60)	1.09 (0.81, 1.46)
1997	0.52 (0.41, 0.65)	1.09 (0.81, 1.47)	0.70 (0.49, 1.00)
2000	0.42 (0.33, 0.54)	1.32 (0.97, 1.81)	0.68 (0.47, 1.00)
2004	0.27 (0.20, 0.36)	2.03 (1.43, 2.88)	0.45 (0.29, 0.70)
2006	0.20 (0.15, 0.28)	1.89 (1.32, 2.71)	0.38 (0.24, 0.60)
2009	0.13 (0.09, 0.20)	1.86 (1.23, 2.80)	0.27 (0.15, 0.46)
*p-value*	<0.001	<0.001	<0.001

Continuous predictors such as maternal education in years are centred on grand mean

CHNS, China Health and Nutrition Survey

### Individual and household socio-economic factors

As shown in Table 4, after adjustment for socio-economic and demographic factors, the risks of three different malnutrition status decreased as children grew up. As expected, gender does affect the distribution of malnutrition among children in rural China. Being a girl appeared to increase the odds of over-nutrition (overweight only including obesity) by 62% (95% CI: 1.35, 19.94), but decrease the risk of under-nutrition (stunting and/or underweight) by 31% compared to being a boy (95% CI: 0.57, 0.84). There was no gender difference in the risk of paradox of stunted overweight between boys and girls.

Having a health insurance appeared to be an important factor that affected the odds of being under-nutrition and paradox of stunted overweight among rural children after adjustment for socio-economic and demographic confounders. More specifically, the odds of suffering from under-nutrition (stunting and underweight) compared with having a normal nutritional status was reduced by an estimated 23% (95% CI: 0.63, 0.94) among children with health insurance, and the odds of suffering from stunted overweight, relative to having a normal nutritional status, was reduced by an estimated 24% (95% CI: 0.56, 1.03). This may indicate that caregivers of children who have health insurance may have stronger health awareness and better nutritional knowledge than those children without health insurance, which can improve children’s under-nutrition and prevent stunted overweight.

Household socio-economic factors including household income per capita and maternal education were significant predictors of childhood malnutrition. Household income appeared to decrease the odds of under-nutrition among children in graded fashion (95% CI: 0.59, 0.90), while it did not affect the risks of over-nutrition and stunted overweight. More specifically, the odds of under-nutrition were reduced by 8% (95% CI: 0.79, 1.07) for children from medium income families and by 27% (95% CI: 0.59, 0.90) for children from highest income families. In addition, maternal education was a protective factor of childhood under-nutrition and stunted overweight. In other words, the risks of under-nutrition and stunted overweight among children can decrease in rural China when their mothers receive more formal schooling.

### Neighbourhood effects, place of residence, and time effects

The level of urbanisation was a significant predictor of paradox of malnutrition among children (*p*<0.05). The highest level of urbanisation was associated with a substantial reduction of 35% (95% CI: 0.40, 1.05) in the risk of the paradox (stunted overweight), while the medium level of urbanisation tended to increase the risk by 34% (95% CI: 0.50, 0.87). Urbanisation did not appear to affect the likelihood of over-nutrition, while it was associated with decreased risk of under-nutrition among children in a graded fashion (*p* = 0.03). Children from less urbanised areas tended to at greater risk of under-nutrition in rural China.

There were significant regional and temporal disparities in the risk of childhood malnutrition. Compared to children from the most undeveloped Southern areas, children from other places were more likely to be over-nutrition and were less likely to be under-nutrition as well as stunted overweight. Children from the most deprived provinces (south) appeared to have the highest risk of being in paradox of malnutrition compared to those from other regions. The risk of being stunted overweight paradox and under-nutrition among children appeared to decrease over time, while the risk of being over-nutrition in general increase compared to the reference 1991.

### Interaction effects: age and gender

**Fig 1:**
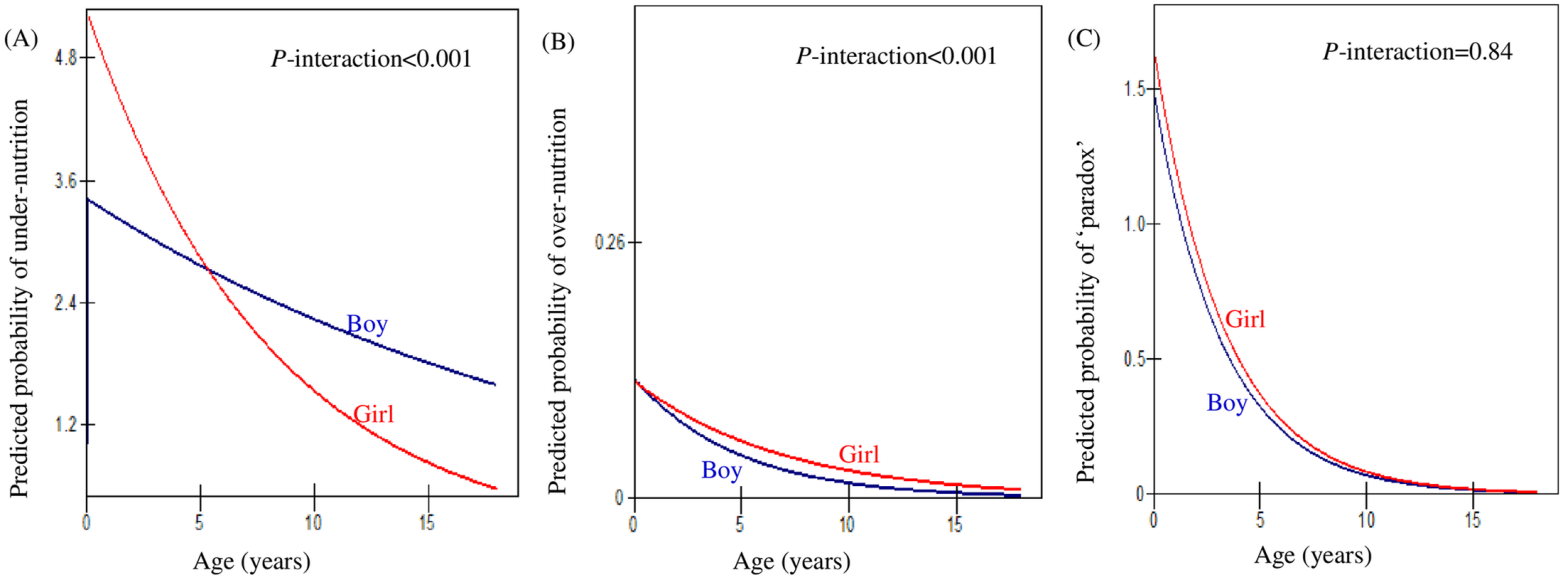
Predicted probability of (A) under-nutrition, (B) over-nutrition and (C) ‘paradox’ of malnutrition (stunted overweight) for rural children less than 18 years old by the interaction of age and gender, CHNS 1991–2009.

In order to facilitate interpretation, Fig 1 was plotted to show the predicted probabilities of under-nutrition, over-nutrition and ‘paradox’ of malnutrition among rural children by the interaction of gender and age fitted by models in Table 5. Increased age was associated with decreased risks of developing all three malnutrition statuses for both boys and girls in rural China. There was little evidence that age modified the association between gender and paradox of malnutrition (stunted overweight) and a joint test of interaction was nonsignificant (*p* = 0.84). However, there was evidence that age modifies the relationships between gender and the risk of under-nutrition (RRR: 0.92; 95% CI: 0.90, 0.95), and the risk of over-nutrition (RRR: 1.06; 95% CI: 1.03, 1.10). Young girls seemed to have higher risk of under-nutrition, and the risk decreased with age more markedly than for boys up to age 5. From age 5 onwards, boys tended to have higher risk of under-nutrition than girls. Girls aged around 12 and older were less likely to suffer from under-nutrition than normal nutrition, while boys of any age appeared to suffer from under-nutrition. Meanwhile, boys and girls were less likely to suffer from over-nutrition compared to normal nutrition at any age. It seems that boys may have an even lower risk of over-nutrition than girls and the gender difference became widened with age until adolescence.

## Discussion

This paper examines patterns, distributions and underlying mechanisms of four exclusive malnutrition status among children in rural China. Household/individual level socio-demographic and socio-economic factors, including gender, only child, child insurance, household income per capita, maternal education are significant predictors of children’s nutritional status in rural China. This study also highlights the importance of contextual factors, urbanisation index and residence of place, and the temporal effects that play a role in children’s malnutrition in rural China. There are substantial regional disparities found in childhood over-nutrition and under-nutrition across China.

China has experienced the double burden of malnutrition among children by adding obesity and related chronic disease to the public health agenda while old nutritional problems of maternal and childhood under-nutrition (stunting and underweight) still remains [11,12,50,51]. This study has made an attempt to explain stunted overweight paradox of children in rural China by using multinomial logistic models with four exclusive scenarios of malnutrition. It has been argued that it is over-simplified to disentangle which common factors contribute to both over-nutrition as well as over-nutrition. We believe our pursuits are rather ambitious because risk factors of child nutrition are complex and can differ according to different status of malnutrition. For example, obesity/overweight among children from developing societies are often driven by improved economic status and purchasing power for energy-dense foods [52,53] and caused by certain contextual factors such as obesogenic physical inactivity environments with urbanisation and adoption of labour-saving devices [54,55]. On the other hand, under-nutrition, such as underweight and stunting, are often associated with inadequate food or nutrient deficiency in relation to deprived economic conditions [56,57]. Despite these differences, it is worth making efforts to explore what factors contribute to the paradox of stunted overweight among children, given that this co-existence can cause even worse consequences among children.

Our results show that improved socioeconomic status, including having health insurance, household income, and maternal education, living in more urbanised villages and residing in more developed areas reduce the risk of stunted overweight among rural children in China. However, children from medium household income families are at increased risk of being stunted overweight. This may suggest that increased income may increase purchasing power for foods but it does not necessarily improve rural children’s stunted overweight status. Evidence shows that household income growth in China can generally improve diet adequacy, but it does not always improve food quality resulting in deficiencies of some key micronutrients [58] and increased consumption of high-fat low-fibre diets [59], especially for low-income families. This may be partly due to poor nutritional knowledge and dietary preferences, as well as food choices available within neighbourhoods.

As show in our study, maternal education and having health insurance are important factors that ought to be considered. There are two possible explanations for their relevance in patterning stunted overweight. First, literacy and numeracy skills gained from education enable the mother to obtain health-related knowledge, for example regarding balanced diets and nutritious foods, and therefore influence children’s nutritional health [60]. On the other hand, caregivers of children who have health insurance may have stronger health awareness and better nutritional knowledge than those of children without health insurance, which can prevent stunted overweight among children in rural China. Important policy implications emerge from these results: improving the household environment, levels of maternal education and living environment from wider context are important to enhance rural children’s nutritional status.

We found that young children less than 3 years tend to be more likely to be stunted overweight than to have a normal nutritional status (Fig 1). Life course epidemiology has suggested that linkages between stunting and obesity are biological in origin and that an infant’s major adaption to undernutrition is a reduced growth rate and related changes in foetal hormone production that cause long-term effects including changes in insulin and growth hormones [61,62]. Evidence suggests that children experience four periods of critical weight gain: the third trimester of gestation, early infancy, period of adiposity rebound (5–7 years) and during adolescence [63]. Children from birth through 3 years of age are most vulnerable to stunting, which may be associated with increased risks of being overweight in later life [20,64,65]. Although the precise age at which stunting relates to the onset of obesity is still unclear [20], it is of critical importance to focus on the early years of life to improve children’s malnutrition status in rural China, as it is elsewhere [66].

Young girls seemed to have a higher risk of under-nutrition than boys and the risk decreased with age when they grow up to around 5 years old, which is consistent with previous evidence [67]. This may suggest that preschool age may constitute a critical period for possible interventions to reduce childhood malnutrition. One possible explanation for gender differences may be due to gender discrimination in favour of boys in intra-household allocation in rural China [36]. There is evidence that girls are more vulnerable than boys to disadvantaged nutrient intakes due to ‘son preference’ norm in rural China [68]. It is well documented that under-nutrition during early childhood restricts children’s physical growth, which may in turn lead to increased risk of dying from infectious disease, or adversely affect cognitive skills, academic achievements, and health and economic capacity in later life [69–73]. We also found that girls aged from 12 onwards were less likely to suffer from under-nutrition, while boys’ higher risk of under-nutrition persisted throughout adolescence. This may suggest that school-aged boys in rural China may be more vulnerable to under-nutrition than their female peers. This is contradicting existing evidence that girls are more disadvantaged in nutritional health than boys in China [67,68]. One possible reason is that biologically, energy requirements differ for boys and girls in order to maintain physical functioning and boys tend be more physically active than girls [74]. Therefore, in case of food scarcity, boys may be more likely to be suffered than girls. Another possible explanation might be due to socio-cultural obligation for boys in rural China that parents tend to save up for son’s adult lives instead of investing in their nutrition, while there is less pressure for girls [75].

In addition, children in rural China were less likely to suffer from over-nutrition compared to normal nutrition at any age. Boys tended to have an even lower risk of over-nutrition than girls and the gender difference became widened with age until adolescence. One possible explanation might be due to sex difference in behavioural factors since boys are generally more physically active than girls especially during adolescence [76,77]. Another possible explanation may be that girls move into pubertal transition earlier than boys [78]and puberty is associated with weight gain [79].

Rapid social and economic change is transforming China with enormous implications for public health. Our study has shown that children from highest urbanised villages are at increased risk of being overweight and obese in rural China. This is consistent with existing evidence that the prevalence of overweight and obesity in children and adolescents increases with the degree of urbanization in China [80]. One possible explanation may be that urbanization is associated with changes in lifestyle and behaviours such as physical inactivity and increased access to high-fat and energy-dense diets. In addition, we found that children from more urbanised areas have reduced risk of under-nutrition in rural China compared to children from lower urbanised villages. It should be noted that the consequences of urbanisation on child health are likely to be mixed. Children may benefit from improved economic status, better access to healthcare, sanitation, and better nutrition. However, rapid environmental, economic and social changes that follow urbanisation may put children at risky situations such as environmental hazards, stressors, and unhealthy diets and lifestyles [81,82]. Therefore, the effect of urbanisation on child nutrition may differ according to the degree of urbanisation and specific socio-cultural contexts.

It should be noted that rural China has been experiencing a large-scale out-migration to urban areas for employment opportunities, which has resulted in about 61 million children living apart from their parent(s) and often under in the care of their grandparents [83]. Studies have found that left-behind children are more vulnerable to malnutrition compared to their counterparts from intact families [34,84,85–87]. Grandparents as caregivers often have poorer nutritional knowledge [88] and beliefs on healthy eating unflavoured for children’s nutritional development [89] than parental caregivers in rural China. Chinese evidence also shows that high rates of malnutrition among children are largely due to a lack of knowledge regarding healthy dietary intake rather than food shortages [7,90]. However, this study did not consider parental migration as the CHNS was not originally designed to study migration. Future studies may benefit from taking into account of parental migration and its role in children’s nutritional health in rural China.

Some methodological limitations warrant cautious interpretations of our findings. First, this study aims to examine risk factors that determine children’s malnutrition status and we have attempted to control for relevant risk factors. There are, however, some key confounders that should be considered but they not available from the secondary dataset—the CHNS, such as birthweight, physical activities and food choices within neighbourhoods which are closely related to children’s nutritional health in rural China. Second, child development can also vary significantly in different periods of life: for example, growth is dramatic in the first two years of life, and then slows down until adolescence [91]. It is plausible to look at different age groups of children as subgroups. For example, children can be divided into two groups: preschool children aged less than 5 years old, and school children aged 6 to 18, because the energy balanced-related behaviours of these two age groups tend to differ [37]. However, sample size restriction in our study discouraged further stratifications by age groups. Future studies can separate children in to different age groups in order to reduce heterogeneity among child population. Third, one of the contextual factors in this study was an urbanisation score, which is a composite measure in relation to population density, transportation infrastructure, sanitation, housing, health infrastructure and some other factors that can distinguish features of urban places [43]. The use of such a composite measure of urbanisation failed to identify specific contextual factors that influence childhood malnutrition. Future studies can take into account of relevant contextual factors to provide specific evidence for policy interventions. Finally, one key limitation in the CHNS, as in any longitudinal dataset, is missing data and sample attrition. This study only included complete cases and this may lead to biased results. For example, older children may drop out from later waves because they may go to boarding schools and subsequently enter colleges and universities. Adolescents may themselves migrate when they are aged over 16 years old [39].

Although we are unable to make definitive statements using the CHNS data—an observational study, our findings highlight that in an urbanised society, a mixture of risk factors from individual, household and neighbourhood levels play their roles in under-and over-nutrition among children in rural China. Our results have important policy implications that improving household environment and maternal education as well as living environment from wider context are important to enhance rural children’s nutritional status in China. Our finding also hightlights that investment in early years of childhood can be effective to reduce gender inequality in nutritional health in rural China.

## Supporting Information

S1 Table Sample sizes (and percentages %) of stunting across overweight/underweight status among children in rural China, the CHNS 1991–2009. (DOCX)

S2 Table Sample sizes (and percentages %) of four exclusive malnutrition categories among children in rural China, the CHNS 1991–2009. (DOCX)
