# Supplementary material for: Patterns and Determinants of Double-Burden of Malnutrition among Rural Children: Evidence from China
Source: PLoS One. 2016 Jul 8;11(7):e0158119. doi: 10.1371/journal.pone.0158119 (PMC4938417; doi:10.1371/journal.pone.0158119)
Supplement: S1 Table — (DOCX) [file pone.0158119.s001.docx]

**S1 Table. Sample sizes (and percentages %) of stunting across overweight/underweight status among children in rural China, the CHNS 1991-2009**

| **Wave** | **Overweight/underweight status** | | | **Total** |
| --- | --- | --- | --- | --- |
|  | **Normal** | **Overweight** | **Underweight** |  |
| ***1991*** |  |  |  |  |
| Not stunted | 1,040 (62.31) | 159 (51.13) | 26 (10.16) | 1,225 (54.79) |
| Stunted | 629 (37.69) | 152 (48.87) | 230 (89.94) | 1,011 (45.21) |
| Subtotal | 1,669 | 311 | 256 | 2,236 |
| ***1993*** |  |  |  |  |
| Not stunted | 1,233 (67.08) | 195 (54.93) | 34 (15.96) | 1,462 (60.76) |
| Stunted | 605 (32.92) | 160 (45.07) | 179 (84.04) | 944 (39.24) |
| Subtotal | 1,838 | 355 | 213 | 2,406 |
| ***1997*** |  |  |  |  |
| Not stunted | 1,094 (71.13) | 141 (64.09) | 30 (22.90) | 1,265 (66.97) |
| Stunted | 444 (28.87) | 79 (35.91) | 101 (77.10) | 624 (33.03) |
| Subtotal | 1,538 | 220 | 131 | 1,889 |
| ***2000*** |  |  |  |  |
| Not stunted | 1,087 (76.23) | 143 (68.10) | 24 (21.62) | 1,254 (71.78) |
| Stunted | 339 (23.77) | 67 (31.90) | 87 (78.38) | 493 (28.22) |
| Subtotal | 1,426 | 210 | 111 | 1,747 |
| ***2004*** |  |  |  |  |
| Not stunted | 790 (81.19) | 166 (77.93) | 18 (23.38) | 974 (77.12) |
| Stunted | 183 (18.81) | 47 (22.07) | 59 (76.62) | 289 (22.88) |
| Subtotal | 973 | 213 | 77 | 1,263 |
| ***2006*** |  |  |  |  |
| Not stunted | 659 (84.38) | 145 (75.92) | 21 (31.34) | 825 (79.40) |
| Stunted | 122 (15.62) | 46 (24.08) | 46 (68.66) | 214 (20.60) |
| Subtotal | 781 | 191 | 67 | 1,039 |
| ***2009*** |  |  |  |  |
| Not stunted | 700 (88.05) | 162 (81.00) | 21 (38.18) | 883 (84.10) |
| Stunted | 95 (11.95) | 38 (19.00) | 34 (61.82) | 167 (15.90) |
| Subtotal | 795 | 200 | 55 | 1,050 |
|  |  |  |  |  |
| **Total** | 9,020 (77.56) | 1,700 (14.62) | 990 (7.82) | 11,630 (100) |

CHNS, China Health and Nutrition Survey
