# Supplementary material for: Patterns and Determinants of Double-Burden of Malnutrition among Rural Children: Evidence from China
Source: PLoS One. 2016 Jul 8;11(7):e0158119. doi: 10.1371/journal.pone.0158119 (PMC4938417; doi:10.1371/journal.pone.0158119)
Supplement: S2 Table — (DOCX) [file pone.0158119.s002.docx]

**S2 Table. Sample sizes (and percentages %) of four exclusive malnutrition categories among children in rural China, the CHNS 1991-2009**

| **Wave** | **Malnutrition** | | | | **Total** |
| --- | --- | --- | --- | --- | --- |
|  | **Normal** | **Under-nutrition**  (stunted and/or underweight) | **Over-nutrition**  (overweight only) | **Paradox**  (stunted overweight) |  |
| 1991 | 1,040 (46.51) | 885 (39.58) | 159 (7.11) | 152 (6.80) | 2,236 |
| 1993 | 1,233 (51.25) | 818 (34.00) | 195 (8.10) | 160 (6.65) | 2,406 |
| 1997 | 1,094 (57.91) | 575 (30.44) | 141 (7.46) | 79 (4.18) | 1,889 |
| 2000 | 1,087 (62.22) | 450 (25.76) | 143 (8.19) | 67 (3.84) | 1,747 |
| 2004 | 790 (62.55) | 260 (20.59) | 166 (13.14) | 47 (3.72) | 1,263 |
| 2006 | 659 (63.43) | 189 (18.19) | 145 (13.96) | 46 (4.43) | 1,039 |
| 2009 | 700 (66.67) | 150 (14.29) | 162 (15.43) | 38 (3.62) | 1,050 |
|  |  |  |  |  |  |
| **Total** | 6,603 (56.78) | 3,327 (28.61) | 1,111 (9.55) | 589 (5.06) | 11,630 (100) |

CHNS, China Health and Nutrition Survey
